# Antibiotic Activity of Superior Class Traditional Chinese Medicine Herbs on *Escherichia coli*

**DOI:** 10.17912/micropub.biology.001279

**Published:** 2025-10-13

**Authors:** Angela Li, Dominic Kassing, Annette Neuman, LaTonia Taliaferro-Smith

**Affiliations:** 1 Division of Natural Sciences, Oxford College of Emory University, Oxford, GA USA; 2 Department of Chemistry, Division of Natural Sciences, Oxford College of Emory University, Oxford, GA USA; 3 Department of Biology, Division of Natural Sciences, Oxford College of Emory University, Oxford, GA USA

## Abstract

*Escherichia coli* has developed resistance to most available antibiotics, creating an urgent need for alternative antimicrobial agents. Traditional Chinese Medicine herbs have historically treated infectious diseases and may offer promising alternatives. We investigated the antibacterial activity of five superior class TCM herbs against *E. coli* using both ethanol extracts and powdered samples. All herbs demonstrated significant zones of inhibition in both forms, suggesting potent antibacterial properties. These findings indicate that TCM herbs warrant further investigation as potential antimicrobial therapies against antibiotic-resistant bacterial infections.

**Figure 1. Antimicrobial activity of traditional Chinese medicine herb extracts and powderized root samples:**
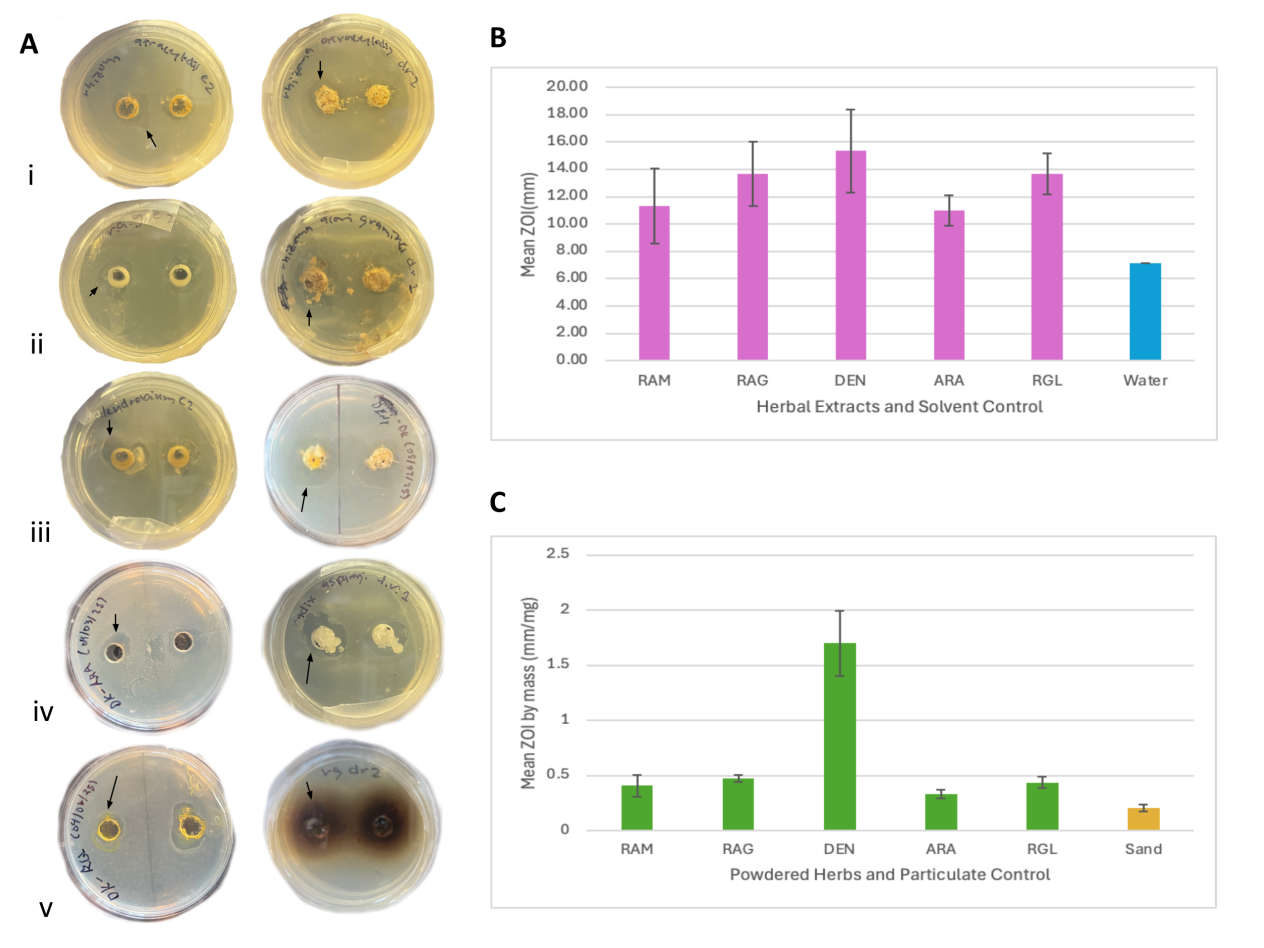
**A) Representative agar well diffusion assay plates.** Results are shown for both herbal extracts (left) and powderized herbs (right) for five TCM herbs: (i) Rhizoma *Atractylodis* *macrocephalae* (RAM), (ii) Rhizoma *Acori graminei* (RAG), (iii) *Dendrobium* (DEN), (iv) *Asparagi radix* (ARA), and (v) raw *Rehmannia glutinosa* (RGL). Zones of inhibition (ZOI) were measured from edge to edge of each transparent zone (arrows indicate zone boundaries). Antimicrobial activity was assessed using six replicate plates (two wells each) for herbal extracts and powderized samples of each herb, plus three control plates each for deionized water (solvent control) and sand (particulate control), totaling 66 plates. **B) Herbal extracts demonstrate significant antimicrobial activity.** All five extracts showed significantly larger ZOIs compared to the deionized water control: RAM (t=3.75, p=0.0038), RAG (t=6.83, p<0.0001), DEN (t=6.66, p<0.0001), ARA (t=8.57, p<0.0001), and RGL (t=10.6, p<0.0001). **C) Powderized herbs demonstrate significant antimicrobial activity.** All five powderized herbs showed significantly larger normalized ZOIs (by mass) compared to the sand control: RAM (t=6.80, p<0.0001), RAG (t=20.1, p<0.0001), DEN (t=17.3, p<0.0001), ARA (t=8.45, p<0.0001), and RGL (t=13.1, p<0.0001).

## Description

Antibiotics have been monumental to modern medicine in preventing bacterial infections and saving millions of lives (Ventola 2015). The overuse and misuse of antibiotics has led to widespread antibiotic-resistant infections, of which gram-negative bacteria like *Escherichia coli* ( *E. coli* ) are becoming resistant to nearly all available antibiotics (Ventola 2015). *E. coli* is commonly found in the gastrointestinal tract and leads to infections characterized by stomach cramps, watery and bloody diarrhea, vomiting, and fever (Mueller and Tainter 2023).

Given the need for more antibiotics to combat antibiotic-resistant infections, we investigated the potential antibiotic properties of Traditional Chinese Medicine (TCM). TCM has a long history of treating infectious diseases, and compared with other antibiotics, TCM has more active ingredients, fewer adverse reactions, and more targets in bacterial cells and walls (Li et al., 2022). Previous studies have found antibiotic properties in various herbs used in TCM (Lal et al., 2020; Wong et al., 2010; Ming et al., 2022; Zhang et al., 2021; Chen et al., 2021; Zhao et al., 2019). TCM classifies herbs into superior class, middle class, and inferior class, of which the superior class herbs have been used as dietary functional foods and possess the least toxicity (Yang 1998, Lee et al., 2012). For this study, five superior class herbs were chosen for their potential antibiotic properties against *E. coli* described in the *Divine Farmer’s Classic of Materia Medica* , a key work in Chinese herbal medicine literature, and results from previous studies (Zhao et al., 2018). Rhizoma *Atractylodis macrocephalae* 白术 (RAM) "treats vomiting and diarrhea" (Yang 1998) and improves digestion (Singh et al., 2024). Rhizoma *Acori graminei* 菖蒲 (RAG) "opens the stomach and eliminates impediment" (Yang 1998) and improves intestinal barrier function (Park et al., 2025). *Dendrobium* 石斛 (DEN) "supplements the stomach, eliminates empty heat and generates fluids" (Yang 1998), and regulates the intestinal microbiota (Li et al., 2022). *Asparagi radix* 天门冬 (ARA) "kills three kinds of worms and removes their hidden corpse" (Yang 1998) and has been used to treat fever and stomachache (Wang et al., 2022). Raw *Rehmannia glutinosa* (生)地黄 (RGL) "eliminates impediments from the heart, kidneys, and intestines" (Yang 1998) and combats intestinal inflammation (Li et al., 2023).

This study investigated the antimicrobial effects of five traditional Chinese medicine herbs—RAM, RAG, DEN, ARA, and RGL—against *E. coli* . The hypothesis tested was that both ethanol extracts and powdered forms of each herb would exhibit antibacterial activity against *E. coli* . The agar well diffusion method was employed to assess antimicrobial activity by measuring zones of inhibition (ZOI) for extracts and mass-normalized ZOI for powdered samples, using deionized water and sand as respective controls ( Figure 1A ).

All five superior class herbs tested possessed significant antimicrobial properties in both herbal extract and powdered forms relative to their respective controls ( Figure 1B, 1C). Among the herbs tested, DEN demonstrated the greatest antimicrobial activity in both extract and powdered forms ( Figure 1B, 1C). DEN exhibited substantially higher mass-normalized ZOI values compared to other powdered herbs and controls, while showing relatively similar mean ZOI to other herbal extracts ( Figure 1B, 1C). This pattern suggests that the particulate matter filtered out during DEN extraction may retain considerable antimicrobial activity. Further investigation is needed to identify the specific antimicrobial compounds present in these five herbs. The active compounds will be isolated and tested in microbial assays to determine their minimum inhibitory concentrations (MICs).

This study demonstrated clear antimicrobial activity of five superior class TCM herbs: rhizoma *Atractylodis macrocephalae* , raw *Rehmannia glutinosa* , rhizoma *Acori graminei* , *Dendrobium* , and *Asparagi radix* . These herbs represent viable candidates for developing new antibiotics against antibiotic-resistant pathogens. This study provides insight into the antibiotic potential of TCM and supports other research highlighting TCM's promise in combating pathogens that contribute to the global antibiotic resistance crisis.

## Methods

*Bacteria Culture* *E. coli* W3104 (Carolina Biological) were preserved under 10°C and were cultured overnight at 37°C in sterilized Luria broth medium (Carolina Biological).

*Herb Obtainment and Preparation*

Herbs were purchased from Tak Yan Herbal in San Jose, California. The maceration method was used for extraction (Abubakar and Haque, 2020). Herbs were ground into powdered form with a sterilized blender. For the extracts, 50g of each powdered herb was extracted in 300mL of 190 proof (95%) non-denatured ethanol ZDQ-ZVZT (Lab Alley) at 23°C for one week using a magnetic stirrer. Both the herbs and storage containers for the herbs were sprayed with ethanol prior to storage and the containers were sealed until use. The insoluble components of the extracts were filtered out with coarse filter paper, and all ethanol was removed by rotary evaporation. 1 gram of ethanol-free pure extract was diluted with 10 mL DI water to create the final liquid extract.

*Microbial Assays*

Agar well diffusion was used to assess antimicrobial activity. 50 µL of bacteria culture was plated on nutrient agar plates (Carolina Biological). Two wells were made in each agar plate using the backs of sterile 200 µL pipette tips. 50 µL of each herbal extract and 40 mg of each powdered herb (10 mg for powdered DEN) were added to each well. Control plates with 50 µL water and 40 mg sand were also prepared. Bacterial plates were incubated at 37°C for 24 hours before analysis.

*Data Collection and Analysis*

The maximum diameter of the ZOI was determined for each herbal sample type by measuring from one edge of each transparent zone to the other edge. The ZOI for the controls, which had very little to no antibiotic effects, were measured as the diameter of the wells. Mean ZOI in mm was used for the herbal extract analysis, and ZOI data for powdered samples were normalized by dividing the inhibition zone diameter (mm) by sample mass (mg) to yield mm/mg values for comparative analysis. Unpaired t-tests were used to assess the significance of the mean ZOI or mean ZOI by mass for each sample compared to their respective controls.

## Reagents

**Table:** 

Strain/Catalog Number	Genotype/Reagent	Available From
W3104	*Escherichia coli*	Carolina Biological
ZDQ-ZVZT	190 proof (95%) non-denatured ethanol	Lab Alley
216710	Luria broth medium	Carolina Biological
785301	Nutrient agar	Carolina Biological
